# The 2023 Impact of Inflammatory Bowel Disease in Canada: Indirect (Individual and Societal) and Direct Out-of-Pocket Costs

**DOI:** 10.1093/jcag/gwad009

**Published:** 2023-09-05

**Authors:** M Ellen Kuenzig, James H B Im, Stephanie Coward, Joseph W Windsor, Gilaad G Kaplan, Sanjay K Murthy, Eric I Benchimol, Charles N Bernstein, Alain Bitton, Jennifer L Jones, Kate Lee, Juan-Nicolás Peña-Sánchez, Noelle Rohatinsky, Sara Ghandeharian, Tyrel Jones May, Sahar Tabatabavakili, Rohit Jogendran, Jake Weinstein, Rabia Khan, Elias Hazan, Mira Browne, Tal Davis, Quinn Goddard, Julia Gorospe, Kate Latos, Kate Mason, Jack Kerr, Naji Balche, Anna Sklar, Laura E Targownik

**Affiliations:** SickKids Inflammatory Bowel Disease Centre, Division of Gastroenterology, Hepatology, and Nutrition, The Hospital for Sick Children, Toronto, Ontario, Canada; Child Health Evaluative Sciences, SickKids Research Institute, The Hospital for Sick Children, Toronto, Ontario, Canada; SickKids Inflammatory Bowel Disease Centre, Division of Gastroenterology, Hepatology, and Nutrition, The Hospital for Sick Children, Toronto, Ontario, Canada; Child Health Evaluative Sciences, SickKids Research Institute, The Hospital for Sick Children, Toronto, Ontario, Canada; Departments of Medicine and Community Health Sciences, University of Calgary, Calgary, Alberta, Canada; Departments of Medicine and Community Health Sciences, University of Calgary, Calgary, Alberta, Canada; Departments of Medicine and Community Health Sciences, University of Calgary, Calgary, Alberta, Canada; Department of Medicine, University of Ottawa, Ottawa, Ontario, Canada; The Ottawa Hospital IBD Centre, Ottawa, Ontario, Canada; SickKids Inflammatory Bowel Disease Centre, Division of Gastroenterology, Hepatology, and Nutrition, The Hospital for Sick Children, Toronto, Ontario, Canada; Child Health Evaluative Sciences, SickKids Research Institute, The Hospital for Sick Children, Toronto, Ontario, Canada; Department of Paediatrics, Temerty Faculty of Medicine, University of Toronto, Toronto, Ontario, Canada; Institute of Health Policy, Management, and Evaluation, Dalla Lana School of Public Health, University of Toronto, Toronto, Ontario, Canada; Department of Internal Medicine, Max Rady College of Medicine, Rady Faculty of Health Sciences, University of Manitoba, Winnipeg, Manitoba, Canada; University of Manitoba IBD Clinical and Research Centre, Winnipeg, Manitoba, Canada; Division of Gastroenterology and Hepatology, McGill University Health Centre IBD Centre, McGill University, Montréal, Quebec, Canada; Departments of Medicine, Clinical Health, and Epidemiology, Dalhousie University, Halifax, Nova Scotia, Canada; Crohn’s and Colitis Canada, Toronto, Ontario, Canada; Department of Community Health and Epidemiology, University of Saskatchewan, Saskatoon, Saskatchewan, Canada; College of Nursing, University of Saskatchewan, Saskatoon, Saskatchewan, Canada; Crohn’s and Colitis Canada, Toronto, Ontario, Canada; Division of Gastroenterology and Hepatology, University Health Network, University of Toronto, Toronto, Ontario, Canada; Department of Gastroenterology, University of Toronto, Toronto, Ontario, Canada; Department of Medicine, University of Toronto, Toronto, Ontario, Canada; SickKids Inflammatory Bowel Disease Centre, Division of Gastroenterology, Hepatology, and Nutrition, The Hospital for Sick Children, Toronto, Ontario, Canada; Child Health Evaluative Sciences, SickKids Research Institute, The Hospital for Sick Children, Toronto, Ontario, Canada; SickKids Inflammatory Bowel Disease Centre, Division of Gastroenterology, Hepatology, and Nutrition, The Hospital for Sick Children, Toronto, Ontario, Canada; Child Health Evaluative Sciences, SickKids Research Institute, The Hospital for Sick Children, Toronto, Ontario, Canada; ICES, Toronto, Ontario, Canada; Department of Internal Medicine, University of Toronto, Toronto, Ontario, Canada; SickKids Inflammatory Bowel Disease Centre, Division of Gastroenterology, Hepatology, and Nutrition, The Hospital for Sick Children, Toronto, Ontario, Canada; Child Health Evaluative Sciences, SickKids Research Institute, The Hospital for Sick Children, Toronto, Ontario, Canada; SickKids Inflammatory Bowel Disease Centre, Division of Gastroenterology, Hepatology, and Nutrition, The Hospital for Sick Children, Toronto, Ontario, Canada; Child Health Evaluative Sciences, SickKids Research Institute, The Hospital for Sick Children, Toronto, Ontario, Canada; Departments of Medicine and Community Health Sciences, University of Calgary, Calgary, Alberta, Canada; Departments of Medicine and Community Health Sciences, University of Calgary, Calgary, Alberta, Canada; Crohn’s and Colitis Canada, Toronto, Ontario, Canada; Crohn’s and Colitis Canada, Toronto, Ontario, Canada; Department of Medicine, Memorial University of Newfoundland, St John’s Newfoundland, Canada; Crohn’s and Colitis Canada, Toronto, Ontario, Canada; Crohn’s and Colitis Canada, Toronto, Ontario, Canada; Division of Gastroenterology and Hepatology, Mount Sinai Hospital, University of Toronto, Toronto, Ontario, Canada

**Keywords:** Absenteeism, Caregiving costs, Crohn’s disease, Lost productivity, Presenteeism, Ulcerative colitis

## Abstract

People living with inflammatory bowel disease (IBD) and their caregivers are faced with indirect and out-of-pocket costs that they would not otherwise experience. These costs impact one’s ability to contribute to the economy to their fullest potential. The indirect costs of IBD in Canada are estimated to be at least $1.51 billion in 2023 and include costs associated with lost productivity resulting from a combination of missed work (absenteeism), decreased workplace productivity (presenteeism), unemployment, premature mortality, and caregiving costs. Unemployment is the largest contributor to indirect costs ($1.14 billion), followed by costs of absenteeism and presenteeism ($285 million). Caregiving costs for children with IBD are estimated to be nearly $58 million. Canadians with IBD also pay $536 million every year for care that is not covered by universal or supplemental private health insurance; this includes allied healthcare (e.g., care provided by psychologists), medication, and other supportive therapy. Combined, the indirect and out-of-pocket costs of IBD in Canada are estimated at more than $2 billion CAD in 2023. This is substantially higher than the estimate of $1.29 billion in Crohn’s and Colitis Canada’s 2018 Impact of IBD report with differences attributable to a combination of rising prevalence, inflation, and the addition of presenteeism and caregiving costs to the total indirect costs.

**Key Points**

1. IBD impacts the ability of people living with the disease (or caring for people living with IBD) from contributing to the economy to their fullest potential.
2. In Canada, the indirect costs of IBD are estimated to be at least $1.51 billion, though this is likely an underestimate.
3. Costs of unemployment are the largest contributor to indirect costs ($1.14 billion), followed by costs of absenteeism and presenteeism ($285 million).
4. Caregiving costs for children with IBD are estimated to be nearly $58 million.
5. Canadians with IBD pay $536 million out of their own pockets to obtain care, medications, and supportive therapy not covered by universal or private health insurance plans.

## Summary of Crohn’s and Colitis Canada’s 2018 Impact of IBD: Indirect Costs of IBD Care

The annual indirect and out-of-pocket costs were estimated at $1.29 billion in 2018. Decreased workplace productivity was reported as a significant contributor to indirect costs, driven by premature retirement ($629 million CAD), absenteeism ($88 million CAD), and premature death ($34 million CAD). Out-of-pocket expenses borne by people living with IBD were estimated at $541 million. Data on the costs of presenteeism, professional advancement, and caregiving were lacking and therefore not included in the estimate of costs in 2018.

## INTRODUCTION

**Figure 1.**
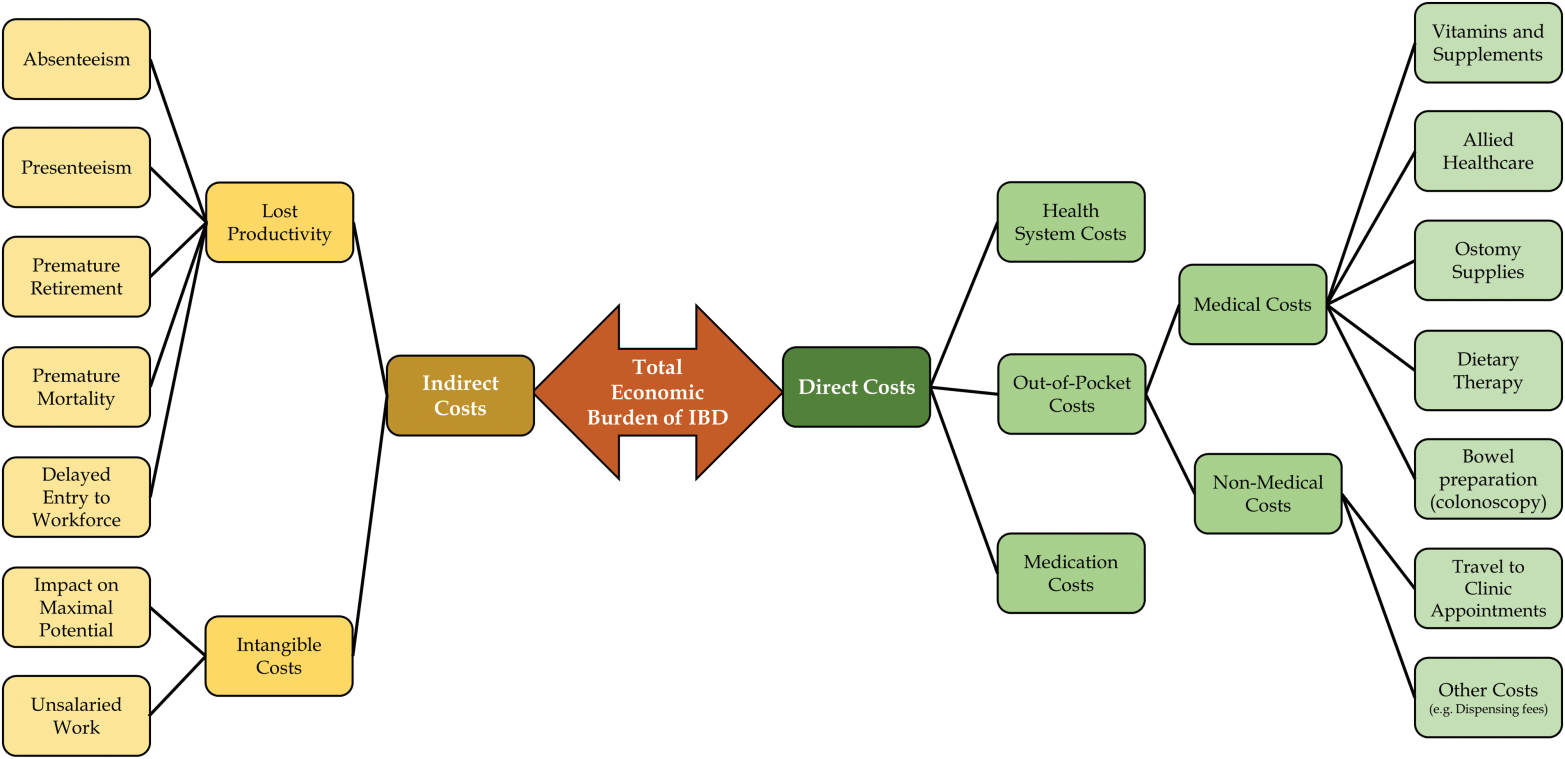
Component costs contributing to the economic burden of inflammatory bowel disease in Canada. Figure adapted from Garaszczuk *et al*. (1).

The total economic burden of the inflammatory bowel diseases (IBD) comprises both direct and indirect costs (Figure 1). Direct costs include those incurred during the course of providing medical care for people with IBD. Direct costs include the costs of clinic visits, emergency department visits, hospitalizations, surgeries, and medications (see Kuenzig *et al.* this volume). In Canada, direct costs are largely born by provincially or territorially administered universal health insurance, supplemented by private extended health benefit plans. However, many people will have to pay for some portion of their direct medical costs out-of-pocket when not covered by governmental or supplemental private insurance, in addition to direct non-medical costs required for individuals to access needed healthcare (e.g., transportation to a clinic appointment). Indirect costs are costs to the individual and society because of the impact that IBD has on a person’s ability to contribute to their full potential. This article details recent data on the indirect and out-of-pocket costs experienced by people living with IBD in Canada.

## INDIRECT COSTS

Indirect costs attributable to a disease reflect the impact that living with a disease (such as IBD) has on a person’s ability to achieve their full potential for engaging with the world. In this sense, indirect costs of IBD can be thought of as lost opportunity. On the level of the individual living with IBD, indirect costs refer to the costs of what a person is missing out on, either in work or in life, because of IBD. On a societal level, indirect costs refer to the loss to society resulting from the impact that IBD has on a person’s ability to fully contribute to their community. Indirect costs are often separated into work-related costs (the loss to the community when a person is unable to work to their full potential) and intangible costs (costs that are difficult to measure, such as the cost to a person from not being able to participate in leisure activities or otherwise enjoy life to their fullest). In addition, the care needs of a person living with IBD can impact the abilities of close family members and friends to contribute to their communities; this is especially true for children, the elderly, and people with severe psychiatric disease or neurocognitive deficits. Therefore, in considering the indirect costs of IBD, the costs incurred by primary caregivers cannot be overlooked.

Indirect costs can often be difficult to quantify precisely. Most attempts to measure indirect costs use the human capital approach, where a person’s lost wages are presumed to represent the costs of lost productivity. Further, there are relatively few studies that have focused on the impact that IBD has on one’s ability to be optimally productive at work or otherwise contribute to the well-being of society, especially in the Canadian setting.

## WORK-RELATED COSTS

### Absenteeism

Absenteeism costs refer to lost wages due to missed work. Costs related to intermittent absences can directly impact an individual’s earnings when they do not have paid sick days or impact employers through costs related to replacing an employee or lost productivity due to an employee’s absence. Among respondents to the Canadian Community Health Survey with IBD, those who were employed missed an average of 1.6 (SD: 4.4) days of work over a three-month period; people without IBD missed an average of 1.0 (SD: 3.5) days of work (2). This equated to an additional 1.1 days (95% CI: 0.7, 1.5) of work missed among people living with IBD at a cost of $270 (95% CI: 163, 377) over three months, after accounting for differences in the probability of employment between people with and without IBD (2). Extrapolating to a full year, people with IBD miss an additional 4.4 days at a cost of $1080 every year compared to people without IBD. Based on an estimated 150,775 employed Canadians with IBD, the attributable indirect cost of missed work among people with IBD is estimated to be $163 million annually. This estimate does not capture additional costs related to presenteeism, delayed entry into the workforce, early retirement, or the failure of individuals to reach their maximum potential.

Several studies have described work-related productivity costs using data from Sweden, where they track healthcare data along with data on access of disability pensions and sick leave pay for the entire population. Sick leave and permanent disability result in 63 days of work missed in people with Crohn’s disease (costing $12,719 USD) and 41 days of work missed in people with ulcerative colitis (costing $8219 USD) per year (3). Between 2006 and 2014, the mean number of days of work missed among persons with Crohn’s disease decreased from 88 days to 61 days (4). People missed more work days at time of surgery or the start of biologic therapy; workplace attendance returned to baseline levels within a median of two months following these events (5). Over the five years following initiation of biologic therapy, workplace attendance was significantly better among individuals continuing on their biologics. Similarly, 44% of people with Crohn’s disease missed some work in the first year following diagnosis, but only 9% experienced total work loss five years after diagnosis (6), likely due to effective therapy.

Among 540 persons in the WORK-IBD cohort from the Netherlands, 18% reported missing work in the last week, and 4% had missed at least 50% of workdays (7). An analysis of a US-based health insurance database suggested the indirect costs related to absenteeism was $5490 USD per year among people with IBD compared to $3322 among people without IBD. Women, people with less education, and those of older working age (40–65 years of age) missed more days of work, and people with moderate-to-severe disease and a history of anxiety or depression were more likely to miss work and accrue higher absenteeism costs (4,7).

### Presenteeism

Presenteeism occurs when an individual is present at work but is unable to work at their full potential. Presenteeism costs estimate the cost associated with this reduction in workplace productivity. Among 762 work-age employed Manitobans with IBD, 37% reported reduced workplace productivity during at least one day in the previous two weeks, with 19% reporting reduced productivity on three or more of the previous 14 days (8). In the WORK-IBD cohort, 50% of persons reported some degree of productivity loss in the previous week, with 16% reporting severe productivity loss (≥50% of their usual productivity) (9). The proportion of persons who reported productivity loss of ≥50% was higher in those with Crohn’s disease (27%) than in those with ulcerative colitis (12%). Fatigue was reported by 71% as the main reason for their reduced productivity and was the most frequently cited contributor to productivity loss. A Finnish study of 320 persons followed at a tertiary IBD centre estimated the annual indirect costs ascribable to presenteeism at €644 per year, €1079 among persons using biologic agents (10). Prior to the COVID-19 pandemic, a Dutch study demonstrated improved presenteeism among people with IBD working remotely (11).

Extrapolating costs estimated in the Finnish study (10) to the Canadian context (adjusting for inflation and purchasing power parities) (12), the total cost of presenteeism among people with IBD is estimated to be $812 (CAD) per person per year, or more than $122 million annually among the 150,775 employed Canadians with IBD (2).

### Impact on Achieving Maximal Educational Potential

A Manitoba study reported no delays in graduating from high school among children with IBD and their non-IBD peers (13). Marks on standardized testing in mathematics and language arts were similar. Data were less clear on whether the impact of IBD in childhood and adolescence influenced choices in higher education and career training as people reached adulthood.

Disease severity may be related to missed days of school and delays in educational progression in high school. In a study of 675 German and Austrian students with IBD, 10.4% reported repeating a year of school; the repetition rate was highest among persons who had missed more than two weeks of school related to IBD, or those who had active IBD symptoms (14). A French study of 104 children and adolescents with IBD reported that they missed 4.8% days of school, with digestive symptoms accounting for a third of missed days and medical appointments and procedures accounting for a quarter of missed days (15). It is important to recognize that survey results may underestimate the true burden of IBD on educational attainment and functioning, as the type of parents who are motivated enough to participate in this research may have characteristics that make them more engaged in supporting their children’s navigation of school challenges while living with IBD.

Importantly, childhood IBD may have a long-term influence on earning potential. In a study using population-based Swedish healthcare data, the annual salary at 30 years of age among individuals who were diagnosed with IBD during childhood was 5.4% (95% CI: 1.8, 9.1) less than healthy controls (16). Children who underwent IBD-related surgery or had extensive hospitalization during childhood earned 16.3% (95% CI: 7.9, 24.7) less at 30 years of age than controls without IBD. Whether this earning disparity resulted from decreased educational attainment or the impact of ongoing IBD-related disability remains unclear.

## CAREGIVER COSTS

Among parents and caregivers of Canadian children with IBD, the mean wages lost annually was $8367 (SD: $11,912); costs were greatest among parents of females and parents with lower educational attainment and income (17). In a survey of 120 primary caregivers of American adults with IBD, of whom 79 were fully or partly employed outside the home, 38% experienced absenteeism within the past week (missing nine hours of work on average), and 57% experienced presenteeism, with a mean decrease in productivity of 22%.

Among individuals reporting higher levels of caregiver burden, 65% and 85% reported absenteeism and presenteeism in the previous week, respectively (18). In a multinational European study of 491 children and their caregivers with newly diagnosed pediatric IBD, workplace productivity of caregivers decreased by 44% around diagnosis, but normalized among caregivers of children whose disease was brought under control (19). This loss in workplace productivity was also correlated to caregiver-reported quality of life. The estimated cost of caregiver absenteeism and productivity loss in the first year following a child’s diagnosis was $7276 USD.

Applying the most recent Canadian estimate of $8367 in indirect costs per child with IBD per year to the estimated 6068 children currently living with IBD in Canada and adjusting for inflation, we estimate the total cost of lost wages among caregivers to be $58 million annually. This figure does not account for lost wages among individuals caring for adults and seniors with IBD and therefore likely underestimates the total indirect costs to caregivers associated with IBD.

### Additional Sources of Indirect Costs

Since the 2018 Impact of IBD in Canada report (20), no additional studies have described the costs of premature retirement or mortality in Canada. Based on data from the Canadian Community Health Survey, people living with IBD are significantly less likely than those without IBD to be employed in the previous three months (70% vs. 80%; adjusted relative risk: 0.92; 95% CI: 0.88, 0.96) (2). With an additional 10% unemployment among people with IBD, we expect 23,124 people with IBD of working age (18–64) to be unemployed for reasons related to their IBD (2). With a mean employment income of $52,928 (21), the attributable indirect costs of unemployment among individuals living with IBD equate to $1.23 billion. This estimate accounts for increases in early retirement and long-term disability among individuals with IBD.

According to data from Statistics Canada, there were 53 deaths with either Crohn’s disease or ulcerative colitis listed as the primary cause of death in 2020 among individuals of working age (20–64 years) (22). Using the average retirement age of 64 years, these 53 individuals will have missed a total of 396 years of future work due to their premature deaths. At an average employment income of $52,928, the cost of premature death in Canada is nearly $21 million. This is likely an underestimate as this estimate does not capture deaths where IBD may have been a contributing factor (but not been the primary cause of death) or that occurred due to complications of IBD (e.g., colorectal cancer), its treatment (e.g., an opportunistic infection in someone on immunosuppression) or other chronic conditions that may be elevated among individuals with IBD (see Shaffer *et al.* this volume, Age-Related Comorbidities).

### Estimating the Total Indirect Costs of IBD in Canada

Incorporating costs of absenteeism, presenteeism, unemployment, premature mortality, and caregiving costs, we estimate the total indirect costs of IBD in Canada to be $1.51 billion. However, this is likely an underestimate of the true indirect costs of living with IBD in Canada as they do not capture costs related to delayed entry into the workforce, failure of individuals living with IBD to reach their maximum educational potential, and caregiving costs for adults and seniors with IBD. Additionally, there may be other intangible costs that cannot be assigned a value that increase the societal costs of IBD.

## DIRECT OUT-OF-POCKET COSTS OF IBD

Non-medical direct out-of-pocket costs include costs that are incurred in the process of obtaining medical care, including the costs of parking during a clinic visit, transportation to a medical appointment, or paying a babysitter to watch children while attending an infusion of a medication. Medical out-of-pocket expenses include anything the individual must pay for directly, including the costs of medications or health services (e.g., allied healthcare professionals such as psychologists or dietitians) not covered by insurance, complementary and alternative medicines (vitamins, supplements, medical cannabis), ostomy supplies, travel to attend appointments, dietary therapy (including exclusive enteral nutrition), and medications for bowel preparation prior to colonoscopy. These costs are a major financial burden for people with IBD (23) and pose barriers to accessing needed care. For example, 55% of people with IBD interviewed in an American study reported cost as the greatest barrier to receiving psychotherapy (24). Financial barriers to care are described in further detail in Mathias *et al.* (this volume). Direct costs borne by the healthcare systems and private insurance plans are discussed in Kuenzig *et al.* (this volume).

A Canadian study reported mean out-of-pocket expenses for 243 children with IBD at $5236 (SD: $6931) (17). Travel costs were the highest cost (mean: $2234, SD: 2598), followed by over-the-counter medications and other health and food products (mean: $1894, SD: $3326) and food purchased during IBD-related appointments (mean: $1285, SD: $2172). Tests not covered by provincial healthcare, allied healthcare providers (e.g., psychologists), phone calls related to IBD care, and childcare were also included in these total out-of-pocket costs. Families with private health insurance were significantly less likely to be in the highest quartile of out-of-pocket costs (OR: 0.28; 95% CI: 0.08, 0.96).

A survey was conducted in 2022 by Crohn’s and Colitis Canada aimed at understanding unmet needs of the IBD community. Respondents reported mean out-of-pocket costs of $1579 (SD: $2017). Among the 797 people with IBD responding to the survey, the highest reported costs were prescription medications (mean: $504, SD: $942), vitamins and other supplements (mean: $268, SD: $524), acupuncture or massage therapy (mean: $158, SD: $420), transportation to and from medical appointments (mean: $153, SD: $358), mental health care (mean: $139, SD: $506), and over the counter medications (mean: $128, SD: $270). Only 20% of respondents reported decreasing transportation costs to and from clinic appointments with the shift to virtual care because of the COVID-19 pandemic.

### Estimating the Total Out-of-Pocket Expenses for IBD in Canada

Using the estimate of $5236 for out-of-pocket costs for IBD among the 6068 children living with IBD in Canada, adjusted for inflation, the total out-of-pocket costs among children with IBD is estimated to be over $36 million. Using the estimate of $1579 for out-of-pocket costs of IBD among the 316,530 adults and seniors living with IBD in Canada, the total out-of-pocket costs among adults with IBD is estimated to be $500 million. Combining the pediatric and adult out-of-pocket costs, we estimate the total out-of-pocket costs for IBD to be approximately $536 million.

## CONCLUSIONS

The indirect and out-of-pocket costs of IBD are substantial: $1.51 billion in indirect costs and $536 million in out-of-pocket costs. Combined, indirect and out-of-pocket costs account for more than $2 billion annually. This cost is a substantial increase compared to the estimated $1.29 billion in indirect and out-of-pocket costs estimated in the 2018 report (20). The addition of costs associated with presenteeism and caring for children with IBD to the indirect costs, combined with the rising prevalence of IBD in Canada and inflation contribute to these rising costs. Despite these additional data, there remain many costs that are inestimable in the Canadian context, and there are gaps in our knowledge of indirect costs (e.g., caregiving for seniors with IBD).

As the prevalence of IBD continues to rise, the costs of reduced productivity among individuals with IBD and their caregivers will continue to climb. Furthermore, the financial burden associated with out-of-pocket costs place an undue burden on individuals and their families. Our healthcare systems need to evolve to minimize the costs borne by those living with IBD and their caregivers. People with IBD need to be better informed of their rights in the workplace (25). Systems need to be put in place to remove barriers for gainful employment for individuals with IBD, ensuring they can reach their potential.

## KNOWLEDGE GAPS AND FUTURE RESEARCH DIRECTIONS

1. We need to identify and evaluate strategies to reduce absenteeism and presenteeism among employed Canadians living with IBD. Similarly, we need to identify and evaluate strategies to support students with IBD throughout their educational journeys, including during elementary, secondary, and post-secondary education.
2. We need to describe the short- and long-term cost benefits (to both individuals and the healthcare systems) of providing multidisciplinary care that does not require individuals to pay out of pocket or rely on private insurance.
3. There is a lack of data around the indirect and out-of-pocket costs borne by those acting as caregivers for adults and seniors with IBD, which needs to be described.
4. Additional data are needed to describe the total out-of-pocket costs for people living with IBD, including data on the costs of supplements, ostomy supplies, travel to medical appointments, and dietary therapy.

## PATIENT & CAREGIVER PERSPECTIVE

For patient partners, this chapter presents the concerning picture of the significant costs of IBD for individuals living with this condition and their family caregivers. IBD is an expensive chronic condition with costs borne by individuals and Canadian society due to missed work, decrease in productivity, unemployment, and out-of-pocket expenses. Stakeholders must consider how to support persons living with IBD and promote innovative and holistic solutions. As individuals living with IBD face significant barriers to thriving financially, workplace support alternatives (e.g., increased sick time or flexible work-from-home options on flare days) and enhanced benefits and services (e.g., tax breaks or free access to mental health care) need to be promoted to help persons living with IBD and to maximize their full potential in terms of education, earnings, and productivity. Children and young adults living with IBD require early support to be empowered to reach their maximum potential. The indirect healthcare costs of family caregivers (e.g., parents of children with IBD) also require the attention of stakeholders to promote strategies that support them. The experiences and insights of individuals with IBD and their families should guide these initiatives. Furthermore, patient partners emphasized the importance of recognizing that individuals living with IBD, and their families, are still dealing with significant out-of-pocket expenses. These issues require the attention, strategizing, and operationalization of different stakeholders to help reduce these costs and diminish the burden of the disease on the mental health of individuals living with IBD and their families.

## POLICY IMPLICATIONS & KEY ADVOCACY OUTCOMES

1. Canadian healthcare should evolve to include multidisciplinary care, including access to mental health professionals, dietitians, and other allied healthcare providers whose care improves the quality of life for people living with IBD. Additionally, extended health benefit providers should be levied to provide additional benefits regarding these costs to reduce overall health expenditures.
2. The costs of supplements, ostomy supplies, and nutritional therapies (e.g., exclusive enteral nutrition) should be covered by universal healthcare programs.
3. Virtual healthcare delivery should be continued beyond the COVID-19 pandemic due to its ability to decrease individual and caregiver costs related to travelling and missed work for medical appointments. Provincial health authorities need to continue remuneration for these services, which decrease indirect and out-of-pocket costs to the individual.
4. Crohn’s and Colitis Canada should advocate for workplace programs designed to increase workplace productivity among people living with IBD, including tools to decrease both absenteeism and presenteeism.
5. Crohn’s and Colitis Canada should advocate to government for mandatory paid sick days for all individuals, in all sectors.
6. There is a currently lack of understanding among employees and employers in the workplace around what individual rights and workplace accommodations are, regarding chronic disease. Advocacy should target this educational deficit.
7. Crohn’s and Colitis Canada should advocate for medical travel tax deduction limits be increased (in accordance with inflation), the broadening the scope of national tax benefits to include chronic diseases (e.g., Disability Tax Credit, Employment Insurance caregiving benefits). Consistent guidelines concerning qualifications for these credits are needed (from year to year, and across provinces).
8. Crohn’s and Colitis Canada should work to partner with social workers, financial/tax experts, and/or lawyers to provide people with IBD financial or legal advice on their rights as citizens with a potentially disabling chronic disease. Practitioners need to understand the financial burden IBD places on an individual and should provide referrals to these experts.

## SUPPLEMENT SPONSORSHIP

This article appears as part of the supplement “The Impact of Inflammatory Bowel Disease in Canada in 2023”, sponsored by Crohn’s and Colitis Canada, and supported by Canadian Institutes of Health Research Project Scheme Operating Grant (Reference number PJT-162393).

## Data Availability

No new data were generated or analyzed in support of this review.
